# EXPLORING THE ACTIVATING EFFECT OF THE CARE ENVIRONMENT OF GREEN CARE FARMS FOR PEOPLE WITH DEMENTIA

**DOI:** 10.1093/geroni/igae098.2042

**Published:** 2024-12-31

**Authors:** Katharina Rosteius, Laura Frissen, Bram de Boer, Andrea Gabrio, Hilde Verbeek

**Affiliations:** Maastricht University, Maastricht, Limburg, Netherlands; Maastricht University, Maastricht, Limburg, Netherlands; Maastricht University, Maastricht, Limburg, Netherlands; Maastricht University, Maastricht, Limburg, Netherlands; Maastricht University, Maastricht, Limburg, Netherlands

## Abstract

Engagement and activity is important for people with dementia for their sense of purpose, and for cognitive and physical stimulation. Aiming to increase engagement and activity, innovative long-term care environments like Green Care Farms (GCFs) reorganize care by including nature, animals and a more home-like environment into daily care. Previous studies have shown that GCF-residents are more active than those of regular care facilities. This study explored the role of the GCF-environment in this activation. Ecological Momentary Assessments were conducted with 152 participants from four Dutch GCFs, combined with cognitive and physical assessments. Residents were observed during mornings, afternoons and evenings, with a total of 4,600 valid observations. Each observation combines activity-, engagement-, as well as place scores, which are mapped on a ground plan of the facility. Analyses showed that residents were actively engaged in an activity in 67.9% of the observations. Residents spent most of their time in inside common areas (54.0%), followed by their own room (24.6%), outside (10.6%) and in communal inside areas outside of their ward (e.g. a café) (7.7%). In relation to passive behavior, active engagement was highest outside (87.1%), as well as in communal areas outside of the ward (81.7%) and inside the ward (81.3%). These results show that areas where residents spend time together have most activating potential and call for a more communal organization of care. Further research should explore what in the environment triggered residents’ engagement in order to react to needs arising in a tailored way.

